# No effect of unemployment on intimate partner-related femicide during the financial crisis: a longitudinal ecological study in Spain

**DOI:** 10.1186/s12889-015-2322-0

**Published:** 2015-09-30

**Authors:** J. Torrubiano-Domínguez, C. Vives-Cases, M. San-Sebastián, B. Sanz-Barbero, I. Goicolea, C. Álvarez-Dardet

**Affiliations:** Public Health Research Group, Department of Community Nursing, Preventive Medicine and Public Health and History of Science, Alicante University, San Vicente del Raspeig s/n, 03080 Alicante, Spain; CIBER of Epidemiology and Public Health (CIBERESP), Barcelona, Spain; Unit of Epidemiology and Global Health, Department of Public Health and Clinical Medicine, Umeå University, Umeå, Sweden; National School of Public Health, Institute of Health “Carlos III”, Madrid, Spain

**Keywords:** Spouse abuse, Economic recession, Mortality

## Abstract

**Background:**

Spain’s financial crisis has been characterized by an increase in unemployment. This increase could have produced an increase in deaths of women due to intimate partner-related femicides (IPF). This study aims to determine whether the increase in unemployment among both sexes in different regions in Spain is related to an increase in the rates of IPF during the current financial crisis period.

**Methods:**

An ecological longitudinal study was carried out in Spain’s 17 regions. Two study periods were defined: pre-crisis period (2005–2007) and crisis period (2008–2013). IPF rates adjusted by age and unemployment rates for men and women were calculated. We fitted multilevel linear regression models in which observations at level 1 were nested within regions according to a repeated measurements design.

**Results:**

Rates of unemployment have progressively increased in Spain, rising above 20 % from 2008 to 2013 in some regions. IPF rates decreased in some regions during crisis period with respect to pre-crisis period. The multilevel analysis does not support the existence of a significant relationship between the increase in unemployment in men and women and the decrease in IPF since 2008.

**Discussion:**

The increase in unemployment in men and women in Spain does not appear to have an effect on IPF. The results of the multilevel analysis discard the hypothesis that the increase in the rates of unemployment in women and men are related to an increase in IPF rates.

**Conclusions:**

The decline in IPF since 2008 might be interpreted as the result of exposure to other factors such as the lower frequency of divorces in recent years or the medium term effects of the integral protection measures of the law on gender violence that began in 2005.

## Background

The world economic crisis that broke out at the end of 2007 severely affected the banking sector. In Spain, banks stopped lending credit to companies and individuals, giving rise to dramatic destruction of jobs. For this reason rates of unemployment rose from 13.9 % in the year 2008 to 26.2 % in 2013 [1]. Furthermore, in the year 2011, the Spanish government began to implement a series of austerity measures that resulted in budget cuts for different regions in Spain [2]. The governments of these regions were charged with applying the measures to their budgets. Due to the level of autonomy of the 17 regional governments, in Spain each region has discretion in applying budget cuts to the budget lines thought to be most convenient [3]. The different types of government and the evolution of unemployment provide for a natural experiment that permits a first assessment in a European country of the impact of unemployment on the health of the population.

It is known that mortality rates in countries facing financial crisis have changed historically with respect to periods of economic growth. Currently, death rates seem to be decreasing in several of these countries in crisis, but causes of mortality behave differently [4–6]. When there are more people unemployed that are not going to work by car or other transport, traffic accidents decrease and, consequently, mortality due to this cause decreases. On the other hand, financial crisis may be related to an increase in psychological problems and suicide rates, which are usually higher during these periods than during other economic periods [7–10]. In 2009, a study based on data from 26 countries in the European Union estimated that every 1 % increase in unemployment was associated with a 0.79 % increase in suicide rates for those under age 65, and with a 0.79 % increase in homicides [11]. However, studies that provide data disaggregated by sex have shown that female homicides and suicides are not associated with economic fluctuations, and changes in the employment rate due to unemployment affect, above all, those sectors traditionally occupied by men in the majority of European countries [12–15]. In Spain, however, according to data from the Labor Force Survey, unemployment rates have increased since 2008 both in men and women [16].

Our hypothesis in this article is that in the current financial crisis period in Spain, the massive destruction of employment for both men and women could favor or promote a change in homicides of women due to intimate partner violence, also known as intimate partner-related femicides (IPF). As has been observed in other studies, when comparing perpetrators of femicide with other abusive men, unemployment appears to be a significant demographic factor even after controlling for other relevant characteristics [17–19]. As other authors have explained based on Connell’s gender-power theory [20], resorting to intimate partner violence and IPF could be ways of demonstrating manhood when other means to demonstrate manhood and sustain ascendancy cannot be fulfilled, such as when high unemployment rates for men hinder the possibilities to fulfill the role of bread winner [21–23]. Female unemployment has also been previously associated with intimate partner violence and high severity abuse due to limiting women’s possibilities to leave their batterers [24–26].

This study aims to explore how the increase in unemployment among both sexes in the different Spanish regions is related to rates of IPF during the current period of financial crisis. Prior studies showed differences in IPF among the different Spanish regions, which shows that it is important to take geographic distribution into account in the analysis of IPF in Spain [27–29].

## Methods

An ecological longitudinal study was carried out in 17 Spanish regions- Autonomous Communities- in the period 2005 to 2013. The autonomous cities of Ceuta and Melilla were excluded from the analysis because they are not comparable with the other regions studied, given that they are autonomous cities and not regions. Two periods were defined, considering 2008 as the year of the beginning of the economic crisis [13]: Period 1, pre-crisis, years 2005 through 2007; and Period 2, crisis, years 2008 through 2013.

The data on the number of IPF were collected from the Federation of Separated and Divorced Women, whose website has collected and published the cases of IPF for ages 15 and over since the year 1998 [30]. Official data on mortality due to IPV (Intimate Partner Violence) provided by the government of Spain do not permit the calculation of mortality rates by age and standardized for the Spanish population for the 17 Spanish regions [1]. We discarded IPV as an outcome variable because we had much better quality data available on IPF. There are no current realistic annual data on the incidence of IPV, since the majority of IPV cases are hidden by women in this situation. Formal police reports of women are the data that best approach the true incidence of IPV, but these are not disaggregated by age nor by geographic region. The use of IPF ensures disaggregated and trustworthy annual data.

The authors confirmed that 100 % of the deaths recorded by official government sources in Spain during the study period appear in the database of the Federation of Separated and Divorced Women. The Federation of Separated and Divorced Women was founded in 1973. Their activities included the defense of equal rights among men and women and recognition of women’s rights in order to eradicate discrimination in Spanish society. The Federation’s web page records all IPF related deaths in Spain. The authors reviewed this information and added cases to their own database. They double-checked later on that the number of annual deaths due to IPF recorded by the Federation equaled the number of deaths published by official sources such as the Ministry of Equality. The only difference between the two sources was that the information recorded by the Federation is disaggregated. Previous studies show similar percentages of overlap between the database of the Federation of Separated and Divorced Women and the National Observatory on Violence against Women of the Spanish government [28, 29, 31].

Unemployment data were obtained from the Labor Force Survey (LFS) conducted by the National Institute of Statistics of Spain (INE). Unemployment data are collected each trimester through questioning a wide representative sample of the Spanish population about their employment situation during the past weeks. This is the most reliable data to measure unemployment in Spain. An unemployed person is defined as “16 years and over who, during the survey week, was without work, was available for work and actively seeking employment” [16]. This study also included in this definition, a person “qualified to perform work” [16]. Foreign population for the different Spanish regions was obtained from the municipal register published on the website of the National Institute of Statistics of Spain (INE). Inclusion in the municipal register provides immigrants with access to public health and education services, both of which were free in Spain at the time of the start of the crisis.

### Variables

The dependent variable was the annual rate of IPF in the 17 Spanish regions adjusted by age. The independent variable considered was the annual unemployment rate (men and women) in the 17 Spanish regions during the study period. The denominator for calculating unemployment rates was the total labor force [16]. Another independent variable was the annual percentage of foreign population in the different Spanish regions. This variable was included due to its relationship to risk of IPF in Spain [32]. The data required for the denominator of annual IPF rates and to calculate annual foreign population rates were obtained from the municipal register published on the website of the National Institute of Statistics of Spain (INE).

Finally, for the multilevel regression study, a dummy variable was created taking the value of 1 in a period of crisis (2008–2013) and 0 otherwise. Thus it is possible to observe the probability of variation in IPF between the two periods.

### Statistical analysis

**Fig. 1:**
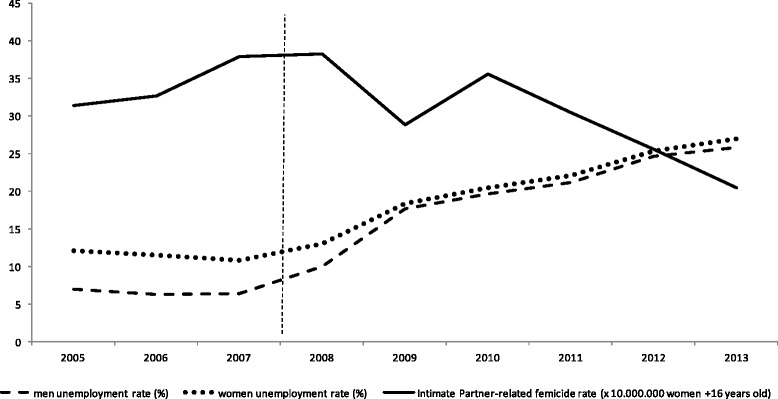
Spain time series for unemployment rates of men and women and intimate partner-related femicide rates (2005–2013)

We calculated crude annual unemployment rates for men and women and age-adjusted rates of Intimate Partner-related Femicide for the years 2005 through 2013 (Fig. 1).

A descriptive analysis was carried out of the distribution of age-adjusted rates of IPF in the 17 Spanish regions. These regional femicide rates were calculated for each year of study by the direct standardization method using the Spanish population as the reference [1]. At the regional level, crude unemployment rates for men and women were calculated for period 1 and period 2. The numerator included the number of unemployed persons in the region and in the period of the study, and the denominator included the labor force population in the region and the period, respectively. Another variable described at the regional level was the percentage of foreign population in the two study periods.

The association between the unemployment rate and the IPF rate was analyzed through multilevel linear regression models. Repeated values of annual unemployment rates and percentages of foreigners formed the first level of the multilevel study. The second level consisted of the 17 Spanish regions, where data on unemployment and immigration are nested. First the null or empty model was adjusted, where only the random part appears. Later, a new multilevel linear regression model with random effects was constructed, including the independent variables described above in the first level: unemployment, foreign population and crisis period.

## Results

A total of 515 cases of IPF took place during the years of the study. In period 1 there were 197 women killed, an average of 65.7 IPF per year. In period 2 there were 318 cases of IPF, an average of 53.0 women killed per year in this period. With respect to unemployment, during the first 3 years of period 1, average unemployment rates for the whole of Spain were 8 %. In period 2, unemployment rates rose to an average of 26.2 % in 2013.

According to the results in Fig. 1, while unemployment rates for both men and women rose, the tendency for the age-adjusted IPF rates during the periods observed was variable, and generally decreased during period 2, which corresponds to the years of crisis studied (Fig. 1).

In period 1, IPF rates of the Spanish regions varied between 0 and 5.61 deaths per million women. In period 2 rates reached a maximum of 7.21 and a minimum of .83 deaths per million women. In ten of the 17 regions studied, IPF rates increased between period 1 and period 2 (Andalucia, Aragon, Canarias, Castilla y Leon, Catalonia, Extremadura, Balearic Islands, Madrid, Galicia and La Rioja). With respect to unemployment data, in period 1 the unemployment rate ranged from 3.79 to 10.20 % for men and from 7.2 to 20.1 % for women. In period 2 the unemployment rate for men ranged from 11.18 to 27.5 %, while for women rates ranged from 12.4 to 30.9 %. Finally, the percentages of foreign population observed in period 1 ranged from 2.11 to 14.11 %. For period 2, these percentages ranged from a minimum of 3.01 % to a maximum of 18.54 % (Table 1).

**Table 1:** Description of IPF (intimate partner-related femicide) rates adjusted by age and unemployment rates and percentage of foreigners in Spain’s regions (2005–2013)

	Period 1 (2005–2007)				Period 2 (2008–2013)			
Spain regions	IPF rates	Percentage of foreigners	Unemployment rates		IPF rates	Percentage of foreigners	Unemployment rates	
			Men	Women			Men	Women
Andalucia	3.37	5.24	9.65	18.3	4.30	7.26	27.22	30.9
Aragón	3.19	7.16	3.84	7.9	3.25	10.56	14.50	16.2
Asturias	4.23	2.43	7.10	12.3	3.46	3.87	16.17	17.7
C. Valenciana	4.40	12.04	6.67	11.4	4.03	14.94	22.47	23.4
Canarias	5.61	10.44	9.24	14.1	7.21	12.81	27.95	28.7
Cantabria	2.58	3.63	5.00	9.7	.83	5.51	13.90	15.0
Castilla y León	2.23	3.57	5.16	12.2	2.81	5.52	14.18	18.8
Castilla La Mancha	4.61	5.95	5.26	13.9	2.50	8.93	19.72	25.8
Cataluña	2.83	10.63	5.56	8.2	3.19	13.05	18.45	17.4
Extremadura	3.85	2.11	10.20	20.1	4.44	3.01	22.20	29.4
Galicia	0.77	2.37	6.32	11.6	1.55	3.38	15.47	16.9
Illes Balears	1.68	14.77	5.39	8.9	2.91	18.54	19.51	19.3
Madrid	2.73	11.62	5.19	8.1	3.16	13.80	15.56	16.0
Murcia	3.98	11.51	5.88	10.8	3.10	13.54	23.24	23.3
Navarra	5.33	7.52	3.79	7.2	1.25	9.11	12.12	13.5
País Vasco	3.22	3.47	5.30	8.8	2.31	5.47	11.18	12.4
La Rioja	0	9.46	4.22	8.6	4.71	11.58	14.11	16.8

The coefficient of the unemployment variable in men in the multilevel linear regression model has a value of −0.069, indicating that for every point increase in unemployment during the 8 years studied, the IPF rate decreased by 0.069 (Table 2). A similar result was observed in the unemployment variable in women, with a regression coefficient value of −0.070. Neither of the unemployment variables is significant at the 95 % confidence interval, but they are significant at 90 % (men unemployed *p*-value = 0.074; women unemployed *p*-value = 0.066). In neither of the two models was the percentage of immigrant population significant, nor was the period of crisis, in explaining IPF (Tables 2 and 3).

**Table 2:** Coefficient estimates, standard errors and deviations for random effects from two level multilevel analysis of Intimate partner-related femicide in 17 Spanish regions 2005–2013 using men unemployed values

	Null model			Model 1. complete model		
Regression components	Coefficient	*p* value	Standard error	Coefficient	*p* value	Standard error
Intercept	2.946	<0.001	0.225	3.248	<0.001	0.603
Men unemployment	--	--	--	−0.069	0.074	0.038
Rate of foreigners	--	--	--	0.052	0.403	0.063
Crisis period (2008–2013)	--	--	--	0.321	0.605	0.619
Variability	Variance component	*p* value	Standard deviation	Variance component	*p* value	Standard deviation
lv. 1 variability	6.369	0.242	2.5237	6.112	0.054	2.472
lv. 2 variability	0.156		0.3946	0.516		0.718

**Table 3:** Coefficient estimates, standard errors and deviations for random effects from two level multilevel analysis of intimate partner-related femicide in 17 Spanish regions 2005–2013 using women unemployed values

	Null model			Model 1. complete model		
Regression components	Coefficient	*p* value	Standard error	Coefficient	*p* value	Standard error
Intercept	2.946	<0.001	0.225	3.629	<0.001	0.664
Women unemployment	--	--	--	−0.070	0.066	0.037
Rate of foreigners	--	--	--	0.051	0.405	0.061
Crisis period (2008–2013)	--	--	--	0.0952	0.859	0.533
Variability	Variance component	*p* value	Standard deviation	Variance component	*p* value	Standard deviation
lv. 1 variability	6.369	0.242	2.5237	6.131	0.084	2.476
lv. 2 variability	0.156		0.3946	0.451		0.672

## Discussion

### Main finding

Over the course of the last 5 years IPF rates in Spain have decreased, while unemployment rates for men and women have increased progressively. The results of the multilevel analysis discard the hypothesis that the increase in the rates of unemployment in women and men, characteristics of the financial crisis in Spain, are related to an increase in IPF rates. This study is a first approximation to this subject; we currently have little information about this issue, and the results could change during the coming years.

### Comparison with previous studies

Prior studies have shown that the possibility of serious abuse of women increases with decreasing income and in families where men are unemployed [25, 33]. Also, male unemployment is associated with a greater risk of IPF [17, 19]. In this study, contrary to what was expected, rates of IPF decreased during a period of crisis (2008–2013) and this tendency does not seem to be related to the increase in unemployment. In interpreting these results it is important to consider the methodological differences that exist between this study and earlier studies. Prior studies on unemployment and IPF and IPV (without a resulting death) are based on individuals and use, as the reference group, abusive men who have not assassinated their partners (IPV) or men who had never mistreated their partners in the past (IPF) [18, 19, 33]. Furthermore, these prior studies do not take into account the time evolution of both indicators (IPF and unemployment). This study is, to our knowledge, the first study concerning IPF in a period of economic change.

There are also other interpretations of these results that should be explored in future studies. One such interpretation could be related to the concept of hegemonic masculinity as described by Connell (1985) [34]. Hegemonic masculinity is not a fixed concept but evolves and adapts to different contexts and times, such that new, less operative ways of being a man become hegemonic or new oppression may emerge [21, 35]. The reduction in IPF rates during the years of the economic crisis could be a reflection of changes in hegemonic masculinity. An explanation of this pattern could be that both men and women try to adapt to the new situation: women by better fulfilling the features of sacrifice and emphasized femininity, and men by avoiding the most extreme forms of violence while resorting to more subtle and chronic forms of IPV.

Additionally, it must be taken into account that in Spain the number of divorces and separations decreased by 19.18 % between 2005 and 2012 [36]. Divorce is commonly identified as a critical moment in the relationship of a couple involved in an IPV situation and can increase the severity of violent acts against women in addition to the probability of death threats both physical and psychological [26, 37]. It could be said, therefore, that some women in situations of IPV have been forced by economic circumstances to “accept” and “bear” the violence of their partners, and this could have had an influence on the general reduction of IPF in Spain. In this case, in contrast to changes in the attitudes of men, the decrease in the rates of IPF does not necessarily imply a reduction in IPV, rather it could imply a continued increase in IPV cases that are hidden [38]. The increase in formal declarations of IPV between 2005 and 2011 according to the 5th annual report of the National Observatory of Violence Against Women in Spain serves as an example [39]. The ability to adapt to changing scenarios without changing the structure of gender power relations is what ensures the pervasiveness of hegemonic masculinity [35, 40].

It is also possible that there is an explanation related to the measures for integral protection against gender violence laid out by Organic Law 1/2004 [41]. In the same way that the increase in unemployment in some countries does not correspond to increases in general mortality due to the modulating effect of social protection policies [5], It could be that the reduction in IPF in recent years that coincide with the period of economic crisis is also a result of the provisions of the protection against IPV set forth in the Law Against Violence Against Women, This law took effect in 2005 and that may have “ameliorated” the negative effects of a progressive increase in unemployment in men and women.

### Limitations

The low number of cases of IPF in years studied may have influenced our results. As has been mentioned in previous studies, there is no established date for the start of the financial crisis in Spain [13]. However, we identified 2008 as the first year of economic crisis as has been done in previous research [11, 13]. The limitations of the ecological design should also be taken into account due to the difficulty in inferring relationships at an individual level and the fact that ecological data contain only marginal observations of the joint distribution of individually defined confounders and outcomes.

## Conclusion

In Spain there has been an increase in male and female unemployment rates as a consequence of the economic crisis, there has also been a decrease in IPF rates. It does not seem that unemployment has influenced the observed tendency of IPF, which should therefore be attributed to exposure to other factors such as the decrease in divorce or the protective effects of the gender violence law in effect prior to the beginning of the crisis. However, the decrease in IPF could be related to an increase in invisible IPV cases. Therefore, it is important to continue this line of research with other studies that contemplate other IPV outcomes such as calls to help lines or filing formal police reports. It would also be interesting to analyze the situation in other countries where the government response to the financial crisis has been different.

## Abbreviations

IPV: Intimate partner violence
IPF: Intimate partner-related femicide
VAW: Violence against women
LFS: Force Survey
INE: National Institute of Statistics of Spain
